# Parallel transmit hybrid pulse design for controlled on‐resonance magnetization transfer in *R*_1_ mapping at 7T

**DOI:** 10.1002/mrm.30333

**Published:** 2024-10-14

**Authors:** David Leitão, Raphael Tomi‐Tricot, Philippa Bridgen, Pierluigi Di Cio, Patrick Liebig, Rene Gumbrecht, Dieter Ritter, Sharon Giles, Joseph V. Hajnal, Shaihan J. Malik

**Affiliations:** ^1^ School of Biomedical Engineering and Imaging Sciences King's College London London UK; ^2^ MR Research Collaborations Siemens Healthcare Limited Frimley UK; ^3^ Centre for the Developing Brain, School of Biomedical Engineering and Imaging Sciences King's College London London UK; ^4^ London Collaborative Ultra high field System (LoCUS) King's College London London UK; ^5^ Guy's and St Thomas' NHS Foundation Trust London UK; ^6^ Siemens Healthcare Erlangen Germany

**Keywords:** $B_{1}^{+}$ inhomogeneity, magnetization transfer, parallel transmit, RF pulse design, ultra high field

## Abstract

**Purpose:**

This work proposes a “hybrid” RF pulse design method for parallel transmit (pTx) systems to simultaneously control flip angle and root‐mean‐squared $B_{1}^{+}$ ($B_{1}^{\mathrm{rms}}$). These pulses are generally only designed for flip angle, however, this can lead to uncontrolled $B_{1}^{\mathrm{rms}}$, which then leads to variable magnetization transfer (MT) effects. We demonstrate the hybrid design approach for quantitative imaging where both flip angle and $B_{1}^{\mathrm{rms}}$ are important.

**Theory and Methods:**

A dual cost function optimization is performed containing the normalized mean squared errors of the flip angle and $B_{1}^{\mathrm{rms}}$ distributions weighted by a parameter λ. Simulations were conducted to study the behavior of both properties when simultaneously optimizing them. In vivo experiments on a 7T MRI system with an 8‐channel pTx head coil were carried out to study the effect of the hybrid design approach on variable flip angle $R_{1}$(= 1/T_1_) mapping.

**Results:**

Simulations showed that both flip angle and $B_{1}^{\mathrm{rms}}$ can be homogenized simultaneously without detriment to either when compared to an individual optimization. By homogenizing flip angle and $B_{1}^{\mathrm{rms}}$, $R_{1}$ maps were more uniform (coefficient of variation 6.6% vs. 13.0%) compared to those acquired with pulses that only homogenized flip angle.

**Conclusion:**

The proposed hybrid design homogenizes on‐resonance MT effects while homogenizing the flip angle distribution, with only a small detriment in the latter compared to a pulse that just homogenizes flip angle. This improved $R_{1}$ mapping by controlling incidental MT effects, yielding more uniform $R_{1}$ maps.

## 1 INTRODUCTION

Ultra‐high field (UHF) imaging (≥7T) offers increased SNR, which can be translated to higher resolutions.^1^, ^2^ However, the higher associated Larmor frequency leads to spatially inhomogeneous RF magnetic ($B_{1}^{+}$) fields,^3^, ^4^ which can severely impact image quality. Several solutions have been proposed to address this^5^, ^6^, ^7^, ^8^ including parallel transmission^7^, ^8^, ^9^, ^10^ (pTx) whereby the RF system is split into multiple independently controlled channels. Optimizing the relative amplitude/phase of every channel to homogenize the created $B_{1}^{+}$ field, known as RF shimming,^11^, ^12^, ^13^ can require large number of channels for full 3D coverage,^11^ therefore, advanced RF pulse designs are usually combined with pTx. Conventional pulse designs^14^, ^15^, ^16^ typically control the nutation of the magnetization for mobile protons (free‐water and fat) that exhibit prolonged transverse magnetization decay times ($T_{2}$), that is, the flip angle produced by an RF pulse, which is based on dynamics described by the Bloch equation.^17^

Biological tissues also contain a pool of “semisolid” magnetization associated with protons in macromolecules^18^ and bound water. These protons typically have very short T_2_ (˜10 μs) making them unobservable to most MRI; their dynamics are also not well‐described by the Bloch equation because they exhibit non‐exponential decays, as captured by the recently proposed generalized Bloch model.^19^ For RF pulses whose durations are significantly longer than the semisolid T_2_ (which is the case for most pulses on human imaging systems), a binary spin‐bath (BSB) formulation^20^, ^21^ provides a good model. According to the BSB model the semisolid magnetization has no transverse magnetization and its longitudinal magnetization is saturated according to the root‐mean‐squared $B_{1}^{+}$ ($B_{1}^{\mathrm{rms}}$) created by the RF pulses. Although in practice the semisolid magnetization is not directly observed, it exchanges magnetization with the free‐water pool, an effect known as magnetization transfer^22^, ^23^ (MT). Hence, RF pulses that produce spatially non‐uniform $B_{1}^{\mathrm{rms}}$ can produce spatially non‐uniform contrast, mediated by MT. The recently proposed parallel transmit pulse design for saturation homogeneity (“PUSH”) method^24^ addressed this effect by controlling the $B_{1}^{\mathrm{rms}}$ distribution produced by the RF pulse. PUSH was applied to design saturation pulses for generation of MT contrast; in this scenario, the direct effect^23^ on the free‐water magnetization, that is, the flip angle, was ignored because the pulses were applied off‐resonance.

In general, most MRI experiments apply pulses on‐resonance, whether for excitation, refocusing, or inversion. Although in most cases one just considers the flip angle of such pulses, they also saturate the semisolid magnetization according to $B_{1}^{\mathrm{rms}}$. This effect is typically smaller than that of the flip angle, nevertheless studies have shown that such “incidental” on‐resonance MT effects are a confound for different sequences including quantitative MT,^25^, ^26^, ^27^, ^28^ single pool relaxometry $R_{1}$ ($=1/T_{1}$) mapping using variable flip angle^28^, ^29^, ^30^, ^31^ (VFA) and inversion recovery^32^ (IR), or even multiple spin echo^33^ (MSE) sequences. In the case of the VFA method, it has been shown^30^ that the apparent relaxation rate (${\hat{R}}_{1}$) varies with the applied $B_{1}^{\mathrm{rms}}$:

(1) $${\hat{R}}_{1}=R_{1}^{f}+k^{\mathrm{fs}}\left(1-\frac{k^{\mathrm{sf}}}{R_{1}^{s}+k^{\mathrm{sf}}+\pi g\left(\Delta \right){\left(\gamma B_{1}^{\mathrm{rms}}\right)}^{2}}\right),$$

where $R_{1}^{f}$ and $R_{1}^{s}$ are the longitudinal exchange rates of the free‐water (f) and semisolid (s) pools, $k^{\mathrm{fs}}$ (and $k^{\mathrm{sf}}$) is the exchange rate from pool f to s (and from pool s to f), g is the semisolid absorption line‐shape at RF frequency offset Δ and γ is the gyromagnetic ratio. The dependence of ${\hat{R}}_{1}$ on $B_{1}^{\mathrm{rms}}$ leads to spatially varying ${\hat{R}}_{1}$ if $B_{1}^{\mathrm{rms}}$ is itself spatially inhomogeneous.

In this work, we propose a generalized “hybrid” RF pulse design method that optimizes both flip angle and $B_{1}^{\mathrm{rms}}$ distributions simultaneously and demonstrate its utility for designing pulses for VFA $R_{1}$ mapping at 7T.

## 2 THEORY

### 2.1 Hybrid design

Conventionally, pTx pulse designs target a desired rotation, or flip angle α, of the free‐water magnetization:

(2) $$\begin{array}{c} \left\{\hat{b},\hat{g}\right\}≔\arg{\min_{b,g}\left\|\alpha \left(b,g\right)-\alpha_{\mathrm{des}}\right\|}_{W}^{2}, \end{array}$$

where b and g are vectors representing the RF and gradient waveforms respectively, $\alpha_{\mathrm{des}}$ is a vector with the desired flip angle, and W is a diagonal matrix with spatial error weightings. We calculate α using the small tip angle (STA) approximation similarly to the spatial domain method from Grissom et al.^34^ Discretizing space in $N_{s}$ samples and time in $N_{t}$ samples, the $N_{s}\times 1$ transverse magnetization vector m is given by:

(3) $$\begin{array}{c} m=\sum_{q=1}^{N_{\mathrm{ch}}}\mathrm{diag}\left(s_{q}\right)A\;b_{q}, \end{array}$$

where $s_{q}$ is a $N_{s}\times 1$ vector with the transmit sensitivities of the $q^{\mathrm{th}}$ channel (transformed into a $N_{s}\times N_{s}$ diagonal matrix), $b_{q}$ is a $N_{t}\times 1$ vector with the time samples of the RF waveform in the $q^{\mathrm{th}}$ channel, and A is the $N_{s}\times N_{t}$ system matrix whose elements are given by:

(4) $$\begin{array}{c} A_{\mathrm{mn}}=i\gamma m_{0}\Delta t\;e^{i\gamma \Delta B_{0}\left(r_{m}\right)\left(t_{n}-\tau \right)}\;e^{ir_{m}\times k\left(t_{n}\right)}, \end{array}$$

where $i^{2}=-1$ is the imaginary unit, $m_{0}$ is the equilibrium magnetization, Δt is the time discretization step, $\Delta B_{0}\left(r_{m}\right)$ is the off‐resonance at coordinates $r_{m}={\left[x_{m}\;y_{m}\;z_{m}\right]}^{T}$, τ is the pulse duration and k is a $3\times N_{t}$ matrix with the excitation k‐space trajectory.^35^ Note that $\alpha \approx |m|/m_{0}$ under the STA, therefore, Eq. (2) targets the magnitude of the magnetization and ignores the excitation phase.

The recently proposed PUSH design instead targets a desired saturation of semisolid magnetization by controlling $B_{1}^{\mathrm{rms}}$ (referred to as β in the optimization):

(5) $$\begin{array}{c} \left\{\hat{b}\right\}≔\arg{\min_{b}\left\|\beta \left(b\right)-\beta_{\mathrm{des}}\right\|}_{W}^{2}, \end{array}$$

where $\beta_{\mathrm{des}}$ is the desired $B_{1}^{\mathrm{rms}}$, and β is given by:

(6) $$\begin{array}{c} \beta_{m}=\sqrt{\frac{1}{\mathrm{TR}}\sum_{n=1}^{N_{t}}\Delta t{\left(\sum_{q=1}^{N_{\mathrm{ch}}}s_{q}\left(r_{m}\right)b_{q}\left(t_{n}\right)\right)}^{2}}, \end{array}$$

where TR is the repetition time. This work considers simple repeating gradient‐recalled sequences where averaging over TR gives the sequence $B_{1}^{\mathrm{rms}}$, although this could be extended to different sequences and pulse types as necessary.

Here, we explore a “hybrid pulse design” method that simultaneously controls α and β using a dual cost function optimization that also includes operational constraints on hardware and specific absorption rate (SAR) for a model subject:

(7) $$\begin{array}{l} \left\{\hat{b},\hat{g}\right\}≔\arg\min_{b,g}\left\{\left(1-\lambda \right)\frac{{\left\|\alpha \left(b,g\right)-\alpha_{\mathrm{des}}\right\|}_{w}^{2}}{{\left\|\alpha_{\mathrm{des}}\right\|}_{w}^{2}}+\lambda \frac{{\left\|\beta \left(b\right)-\beta_{\mathrm{des}}\right\|}_{w}^{2}}{{\left\|\beta_{\mathrm{des}}\right\|}_{w}^{2}}\right\}\\ \begin{array}{ll} s.t. & \begin{array}{l} \mathrm{SA}R_{\text{global}}\left(b\right)\leq \mathrm{SA}R_{\text{global},\max},\\ \mathrm{SA}R_{10g,v}\left(b\right)\leq \mathrm{SA}R_{10g,\max},1\leq v\leq N_{\mathrm{VOP}},\\ \;{𝒫}_{q}\left(b\right)\leq {𝒫}_{\max},1\leq q\leq N_{\mathrm{ch}},\\ \left|b_{q}\right|\leq V_{\max},1\leq q\leq N_{\mathrm{ch}},\\ \begin{array}{l} \left|g\right|\leq G_{\mathrm{amp}}^{\max},\\ \left|\frac{dg}{\mathrm{dt}}\right|\leq G_{\text{slew}}^{\max}. \end{array} \end{array} \end{array}, \end{array}$$

where $\mathrm{SA}R_{\text{global},\max}$ is the global SAR limit, $\mathrm{SA}R_{10g,\max}$ is the 10 g local SAR limit, ${𝒫}_{\max}$ is the maximum average power, $V_{\max}$ is the maximum RF voltage per channel, $G_{\mathrm{amp}}^{\max}$ is the maximum gradient amplitude and $G_{\text{slew}}^{\max}$ is the maximum slew rate. Local SAR is calculated using a virtual observation point (VOP) compressed model^36^ with $N_{\mathrm{VOP}}$ total observation points. The proposed design consists of one term that is the normalized mean squared error of the flip angle weighted by 1−λ and another that is the normalized mean squared error of the $B_{1}^{\mathrm{rms}}$ weighted by λ.

Compared to the more conventional pulse design (Eq. [2]) that usually just targets a desired flip angle $\alpha_{\mathrm{des}}$, the hybrid (HY) design (Eq. [7]) introduces two new parameters: λ and $\beta_{\mathrm{des}}$. The balancing parameter λ allows adjustment to a flip angle only (FA) optimization (λ=0) or to a $B_{1}^{\mathrm{rms}}$ only (i.e., PUSH) optimization (λ=1). We explore HY optimizations where λ∈(0,1). In these cases, λ trades‐off between the flip angle and $B_{1}^{\mathrm{rms}}$ errors in what is called the Pareto front, and its choice depends on the acceptable error for each one.

### 2.2 Lower bound $\beta_{\min}$

The properties α and β from an RF pulse are related, so when choosing targets $\alpha_{\mathrm{des}}$ and $\beta_{\mathrm{des}}$ in Eq. (7) for λ∈(0,1) it is important to ensure that both targets are consistent. Consider an RF pulse applied on‐resonance without any gradients or off‐resonance and with duration τ and waveform $B_{1}^{+}\left(t\right)$; its flip angle is:

(8) $$\begin{array}{c} \alpha =\gamma \int_{0}^{\tau }B_{1}^{+}\left(t\right)\mathrm{dt}={\gamma \;B}_{1}^{\text{peak}}\;\tau \underset{p_{1}}{\underbrace{\int_{0}^{1}b\left(t^{\prime }\right){\mathrm{dt}}^{\prime }}}={\gamma \;B}_{1}^{\text{peak}}\;\tau \;p_{1}, \end{array}$$

where $B_{1}^{\text{peak}}$ is the peak $B_{1}^{+}\left(t\right)$ and $b\left(t^{\prime }\right)$ is the RF waveform normalized to unit duration and amplitude. At the same time the $B_{1}^{\mathrm{rms}}$ produced by this pulse is:

(9) $$\begin{array}{c} \beta =\sqrt{\frac{1}{\mathrm{TR}}\int_{0}^{\tau }{\left|B_{1}^{+}\left(t\right)\right|}^{2}\mathrm{dt}}=\sqrt{\frac{\tau }{\mathrm{TR}}\;|B_{1}^{\text{peak}}{\;|}^{2}}\\ \;\;\times \;\underset{p_{2}}{\underbrace{\sqrt{\int_{0}^{1}{\left|b\left(t^{\prime }\right)\right|}^{2}{\mathrm{dt}}^{\prime }}}}=\sqrt{\frac{\tau }{\mathrm{TR}}\;}B_{1}^{\text{peak}}p_{2}, \end{array}$$

Combining Eqs. (8) and (9) establishes a relationship between the two:

(10) $$\begin{array}{c} \alpha =\frac{\gamma p_{1}\sqrt{\mathrm{TR}\;\tau \;}}{p_{2}}\;\beta . \end{array}$$

Equivalence is only met if $b\left(t^{\prime }\right)$ is real (i.e., pulse is on‐resonance), and there are no off‐resonance and/or applied gradient fields. In the more general case, magnetization can nutate back and forth between the longitudinal axis and transverse plane because of the aforementioned effects such that the final achieved flip angle is less than that given by Eq. (10), resulting in the following inequality:

(11) $$\begin{array}{c} \alpha \leq \frac{\gamma p_{1}\sqrt{\mathrm{TR}\;\tau \;}}{p_{2}}\;\beta . \end{array}$$

Therefore, a minimum $B_{1}^{\mathrm{rms}}$ is needed to achieve any target $\alpha_{\mathrm{des}}$:

(12) $$\begin{array}{c} \beta_{\min}=\frac{p_{2}}{\gamma p_{1}\sqrt{\mathrm{TR}\;\tau }}\alpha_{\mathrm{des}}. \end{array}$$

For any design problem it will be necessary to choose $\beta_{\mathrm{des}}\geq \beta_{\min}$ to ensure that a feasible solution exists.

## 3 METHODS

### 3.1 Simulations

To explore the proposed design, excitation pulses were designed offline using the k_T_‐points^14^ method (5 RF rectangular sub‐pulses of 200 μs duration and 100 μs gradient blips) for a variety of scenarios: all combinations from $\alpha_{\mathrm{des}}$ = 15°, $\beta_{\mathrm{des}}\in \left[0.5\beta_{\min},4\beta_{\min}\right]$ in steps of $0.1\beta_{\min}$ ($\beta_{\min}$ = 0.415 μT), and λ∈[0,1] in 51 logarithmically spaced steps (denser in the bounds and sparser in the middle) were optimized. These optimizations used 3D brain transmit maps from an 8‐channel pTx system (details below) and were solved using a multi‐start strategy with 100 random seeds on a desktop computer (Intel i9‐10 900X @ 3.70 GHz, 64 GB of RAM, 16 cores) with MATLAB R2020b (The MathWorks). The solutions were analyzed in terms of their normalized root mean square error (NRMSE) for α and β.

For comparison, the circular polarized (CP) mode and RF shimming solutions for $\alpha_{\mathrm{des}}$ = 15° were also calculated and analyzed.

### 3.2 Experiments

All experiments were performed using a 7T scanner (MAGNETOM Terra, Siemens Healthcare) in prototype research configuration, with an 8Tx/32Rx head coil (Nova Medical).

#### 3.2.1 Dual Flip Angle $R_{1}$ mapping

The proposed design was used to optimize excitation pulses for $R_{1}$ mapping in a dual flip angle (DFA) experiment.^37^ This application was considered because of its sensitivity to incidental MT effects (Eq. [1]), such that the $B_{1}^{\mathrm{rms}}$ distribution is expected to affect the spatial distribution of ${\hat{R}}_{1}$.

The DFA method is a special case of VFA where only two spoiled gradient echo images (SPGR) are acquired, here, with flip angles $\alpha_{\mathrm{des}}$ = {3°, 15°} (optimal for $T_{1}\approx$ 1300 ms and TR = 8 ms^38^). When holding TR constant, as done here, the Ernst equation can be linearized and $R_{1}$ fitting can be performed via a voxelwise linear regression:

(13a) $$\begin{array}{c} y=mx+b\equiv \frac{s_{\text{SPGR}}}{\sin\alpha }=E_{1}\frac{s_{\text{SPGR}}}{\tan\alpha }+M_{0}\left(1-E_{1}\right), \end{array}$$

(13b) $$\begin{array}{c} {\hat{R}}_{1}=-\frac{\log\left(m\right)}{\mathrm{TR}}, \end{array}$$

where $m=E_{1}=\exp\left(-\mathrm{TR}\times R_{1}\right)$ is the slope of the linear regression, $s_{\text{SPGR}}$ are the measured magnitude SPGR signals and α are the flip angles. Voxelwise fitting was performed using α calculated from a Bloch simulation of the RF pulses and applying the incomplete spoiling correction proposed by Baudrexel et al.^39^

**FIGURE 1:**
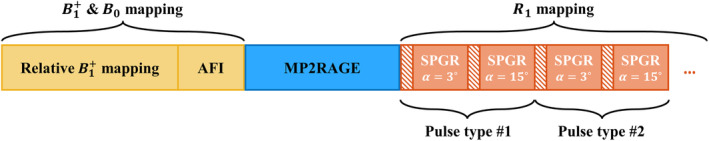
Protocol for in the in vivo experiments, chronologically from left to right. First (in yellow), $B_{1}^{+}$ and $B_{0}$ mapping were performed, with a series of small flip angle spoiled gradient echo images (SPGRs) for relative $B_{1}^{+}$ maps of all transmit channels, and a quantitative $B_{1}^{+}$ and $B_{0}$ map obtained with a multi‐echo actual flip angle imaging (AFI) acquisition. Second (in blue), an MP2RAGE was acquired to provide an independent anatomical reference. Last (in orange), $R_{1}$ mapping was performed for different pulse types, consisting of acquiring two SPGRs (*α* = {3°, 15°}) for each pulse type. The dashed area represents the time spent performing the pulse design, which was done on the scanner for each SPGR before its acquisition.

Six healthy volunteers (25–36 years old, 2 males) were scanned in accordance with local ethical approval. The SPGR volumes (sagittal orientation, $\mathrm{FO}V_{\mathrm{AP}\times \mathrm{RL}\times \mathrm{FH}}$ = 240 × 240 × 192 mm^3^, resolution $1\times 1\times 1\;mm^{3}$, TR = 8 ms, TE = 3 ms, bandwidth =500Hz/Px), GRAPPA^40^ acceleration factor of 2×2 with 32×32 (PE × SL directions, respectively) reference lines were acquired with 20 s of dummy pulses to reach steady state and stabilize the RF power amplifier output,^24^, ^41^ resulting in $T_{\mathrm{acq}}$ = 1 min 59 s per volume. All images were registered using FSL FLIRT^42^ before $R_{1}$ fitting and an additional MP2RAGE^43^ acquired at the same resolution was used for segmentation with SPM12.^44^ $R_{1}$ mapping was repeated using different pulse types as described below, consisting of acquiring the two flip angle SPGRs for each pulse type (Figure 1).

#### 3.2.2 Excitation pulse and data analysis

Before the pulse design, $B_{1}^{+}$ and $B_{0}$ mapping was performed using a combination of a quantitative actual flip angle imaging^45^, ^46^ (AFI) map ($T_{\mathrm{acq}}$ = 2 min18 s; sagittal orientation, $\mathrm{FO}V_{\mathrm{AP}\times \mathrm{RL}\times \mathrm{FH}}=240\times 240\times 240\;mm^{3}$, resolution $5\times 5\times 5\;mm^{3}$, ${\mathrm{TR}}_{1/2}$ = 25/125 ms, α = 60°, TE = {0.75, 1.75, 10}ms, bandwidth =1000Hz/Px, GRAPPA^40^ acceleration factor of 2 with 12 (phase encoding direction) reference lines, slice partial Fourier 6/8, elliptical scanning shutter, 10 s of dummy pulses) and relative per channel estimates via low flip angle SPGR images^47^ ($T_{\mathrm{acq}}$ = 45 s each; sagittal orientation, $\mathrm{FO}V_{\mathrm{AP}\times \mathrm{RL}\times \mathrm{FH}}=240\times 240\times 224\;mm^{3}$, resolution $4\times 4\times 4\;mm^{3}$, TR = 10 ms, TE = 1 ms, bandwidth =1000Hz/Px, 10 s of dummy pulses), as per Figure 1. These maps were then used in the pulse design as described in Section 3.3, before acquiring data for the DFA experiment with the appropriate pulses; pulse calculation was fully scanner‐integrated within a MATLAB framework (R2012b) from the scanner vendor (release Syngo.MR VE12U‐SP01). The optimization was solved using a multi‐start strategy with 10 random starts on the system's console (Intel Xeon E5‐1620 v3 @ 3.5G Hz, 32 GB of RAM, 4 cores), taking ≈20 s.

The DFA $R_{1}$ mapping experiment was conducted using three types of excitation pulses: (1) CP pulse; (2) flip angle optimized pulse with λ=0, referred to as FA pulse; (3) HY optimized pulse with λ=0.5 and $\beta_{\mathrm{des}}=1.2\beta_{\min}$, referred to as HY pulse.

Their implementation is described in Section 3.3. For all six subjects an $R_{1}$ map was acquired using the above three types of excitation pulses. Additionally, for four of these subjects a test–retest of $R_{1}$ mapping was performed with the FA and HY pulses. For the retest, the pulses were redesigned by performing a new optimization from the same $B_{1}^{+}$ and $B_{0}$ maps, but using a different set of random starting points.

The $R_{1}$ maps and their distributions in white matter (WM), gray matter (GM) and CSF were compared across all subjects and pulse types. For a voxel‐based analysis, all $R_{1}$ maps were warped into a common Montreal Neurological Institute (MNI) space using DARTEL^48^ as implemented in the hMRI toolbox,^49^ and the coefficient of variation (CoV) across subjects was calculated for each pulse type. Furthermore, the test–retest $R_{1}$ map acquisitions were compared to assess reproducibility.

### 3.3 Pulse design implementation

The design in Eq. (7) was performed for the excitation pulse types described in Subsection 3.2.2. The CP pulse consisted of a single rectangular RF pulse 200 μs long, whose amplitude was optimized to minimize the flip angle mean squared error with respect to the target. The FA and HY pulses consisted of 5 k_T_‐points^14^ as in the simulations (200 μs RF rectangular sub‐pulses and 100 μs gradient blips), resulting in $\beta_{\mathrm{des}}=1.2\beta_{\min}$ = {0.08 μT, 0.42 μT} for the HY pulses with $\alpha_{\mathrm{des}}$ = {3°, 15°}, respectively. The optimization was solved using a multi‐start strategy in MATLAB with the interior‐point algorithm from the *fmincon* routine, providing first and second order analytical derivatives, and the following constraints: $\mathrm{SA}R_{10g,\max}=20W/\mathrm{kg}$ (using vendor provided SAR model with $N_{\mathrm{VOP}}=8$) in first level SAR mode^50^; $V_{\max}=207V,{𝒫}_{\max}=24W$, $G_{\mathrm{amp}}^{\max}=30\;\mathrm{mT}\;m^{-1}$, $G_{\text{slew}}^{\max}=80\;T\;m^{-1}s^{-1}$. To speed‐up the optimization, the number of voxels was compressed using k‐means clustering similarly to Tomi‐Tricot et al.^51^ Voxels inside a brain mask (obtained using FSL BET^52^) were classified according to their transmit sensitivities, off‐resonance and position, in a total of 500 clusters. The centroid of each cluster was then used as representative of the cluster properties, and the cluster size was used in the weighting matrix W (Eq. (7)).

## 4 RESULTS

### 4.1 Simulations

**FIGURE 2:**
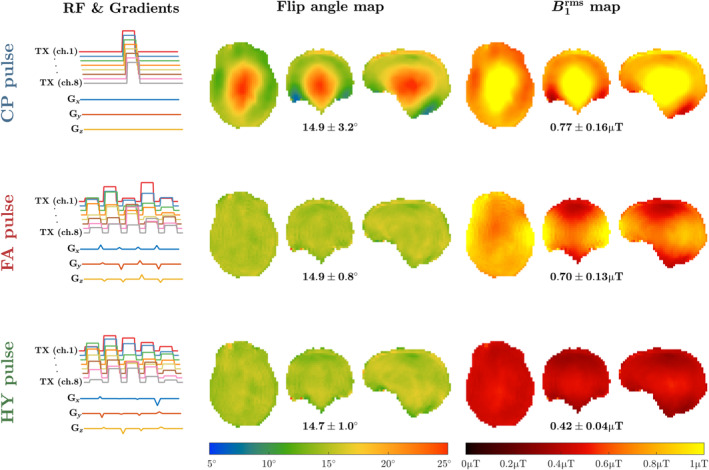
Left column: transmit radiofrequency amplitude and gradients for the considered pulse types (top row: circular polarized (CP) mode pulse; middle row: flip angle optimized k_T_‐points pulse; bottom row: hybrid [HY] optimized k_T_‐points pulse). Their resulting flip angle and $B_{1}^{\mathrm{rms}}$ maps are shown in the middle and right columns, respectively, with the average ± SD of each property depicted below. Target flip angle 15° in all cases, target $B_{1}^{\mathrm{rms}}$ 0.42 μT for the HY pulse (*λ* = 0.5).

Figure 2 shows the flip angle and $B_{1}^{\mathrm{rms}}$ maps achieved with the different excitation pulses. As expected, the CP pulse has a central brightening inhomogeneity in both properties, whereas FA pulse has a very uniform flip angle map, but inhomogeneous $B_{1}^{\mathrm{rms}}$ distribution. When using HY pulse both flip angle and $B_{1}^{\mathrm{rms}}$ become uniform. Figure S1 shows that the maps obtained with a magnitude least squares RF shim are very similar to those of the CP pulse.

**FIGURE 3:**
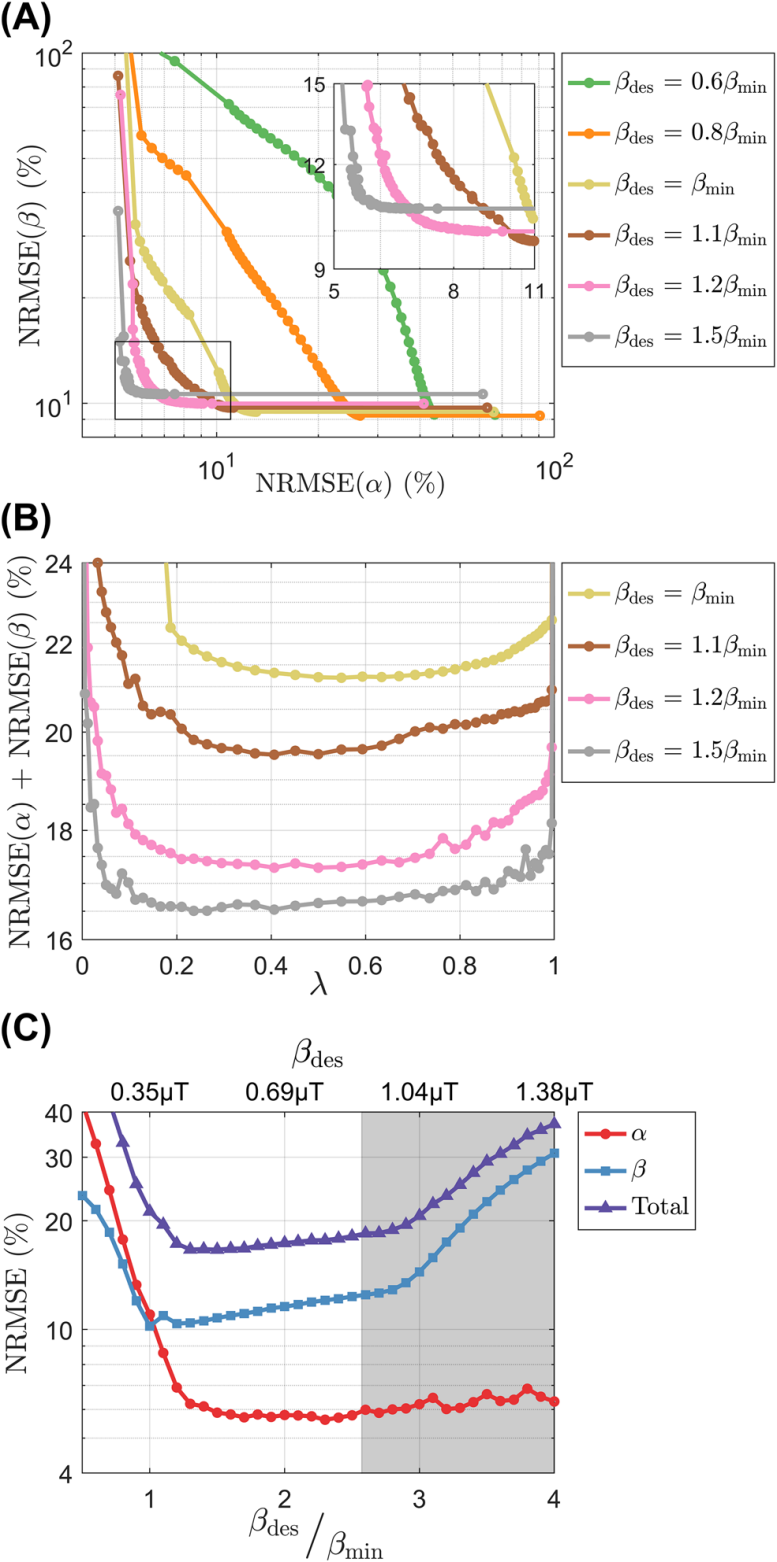
(A) Pareto fronts of the proposed dual cost function for several targets $\beta_{\mathrm{des}}$, including a blown‐up subfigure on the top right corner. (B) Total normalized root mean square error (NRMSE) as a function of λ for several targets $\beta_{\mathrm{des}}$. (C) α, β, and total NRMSE as a function of $\beta_{\mathrm{des}}$ (*x*‐axis on top) and $\beta_{\mathrm{des}}/\beta_{\min}$ (*x*‐axis on bottom) for λ=0.5; the gray area represents unfeasible targets where the circular polarized (CP) pulse would violate the specific absorption rate (SAR) limits.

Figure 3A shows the Pareto fronts representing the trade‐off between the two terms of the proposed cost function (Eq. [7]). For $\beta_{\mathrm{des}}<\beta_{\min}$ the errors in α and β increase substantially when λ≠{0,1} (see Figure 3B), but for $\beta_{\mathrm{des}}\geq \beta_{\min}$ the Pareto front resembles the L‐curve from regularization problems with most solutions located in the corner. This clustering of solutions occurs as the total NRMSE becomes practically constant for λ∈(0,1) as can be seen in Figure 3B. Moreover, as $\beta_{\mathrm{des}}$ increases up to $1.2\beta_{\min}$, the total NRMSE decreases driven by lower NRMSE (α) and then plateaus until $\beta_{\mathrm{des}}$ approaches 1 μT where it reaches SAR limits and increases the NRMSE of β, as can be seen in Figure 3C. Figure S2 expands the visualization of the cost function by plotting the NRMSE of α and β for all $\beta_{\mathrm{des}}$ and λ.

**FIGURE 4:**
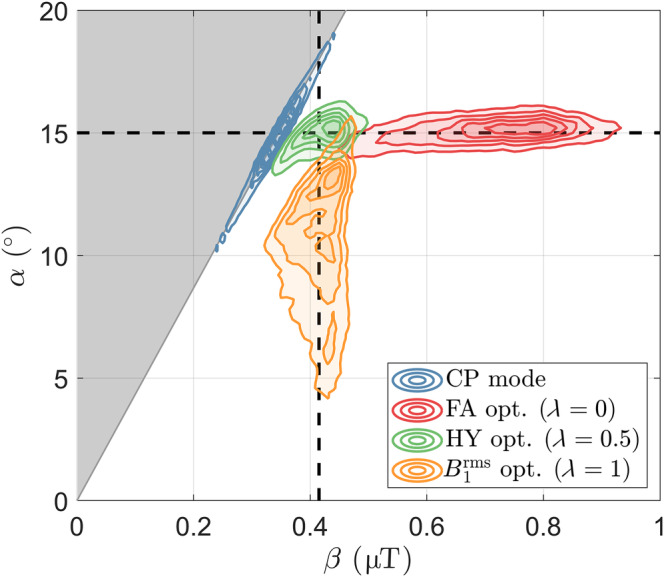
Contours of the flip angle α and root‐mean‐squared $B_{1}^{+}$ β spatial distributions for several pulses: circular polarized (CP) pulse and pTx pulses optimized with λ={0,0.5,1} representing a flip angle (FA) optimization (λ=0), a hybrid (HY) optimization (λ=0.5) and a $B_{1}^{\mathrm{rms}}$ optimization (λ=1). The gray area represents unfeasible points according to Eqs. (11) and (12), whereas the boundary represents the relationship between α and β valid for any static RF shimming solution (including CP mode; Eq. [10]). The distribution contours were slightly smoothed to aid visualization. The dashed black lines depict the two targets $\alpha_{\mathrm{des}}$ = 15° and $\beta_{\mathrm{des}}$ = 0.42 μT (Eq. [7]).

Figure 4 shows the distributions of α and β for the excitation pulses studied. The distribution for CP pulse lies on the line defined by Eq. (10) showing large dispersion in both α and β, which are perfectly correlated because one is proportional to the other in this case. The FA pulse (λ=0) produces a narrow distribution of α, but wide distribution of β because the latter is not controlled by the calculation. Moreover, the $B_{1}^{\mathrm{rms}}$ optimized pulse (λ=1) has a narrow distribution of β, but wide distribution of α. The HY pulse (λ=0.5) achieves both narrow α and β distributions.

### 4.2 Experiments

**FIGURE 5:**
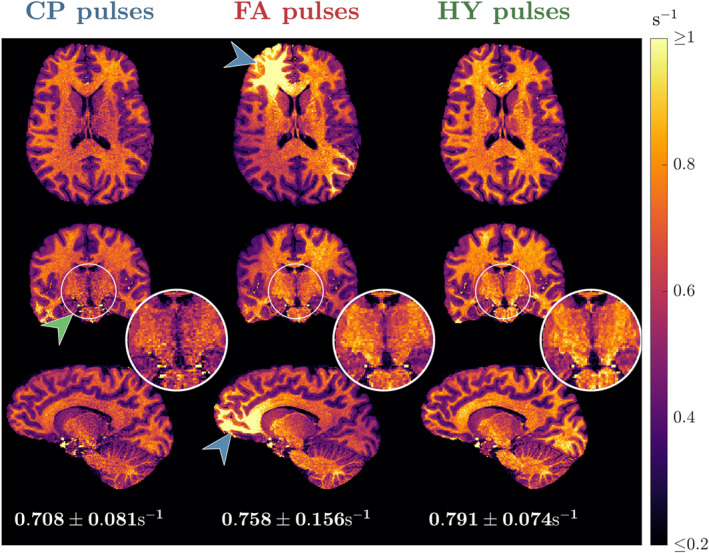
Transverse, coronal and sagittal slices of the ${\hat{R}}_{1}$ maps from subject B using circular polarized (CP) pulses (left column), flip angle (FA) pulses (middle column) and hybrid (HY) pulses (right column). Below each sagittal slice is the average ± SD of ${\hat{R}}_{1}$ in white matter (WM) for the respective pulse type.

**FIGURE 6:**
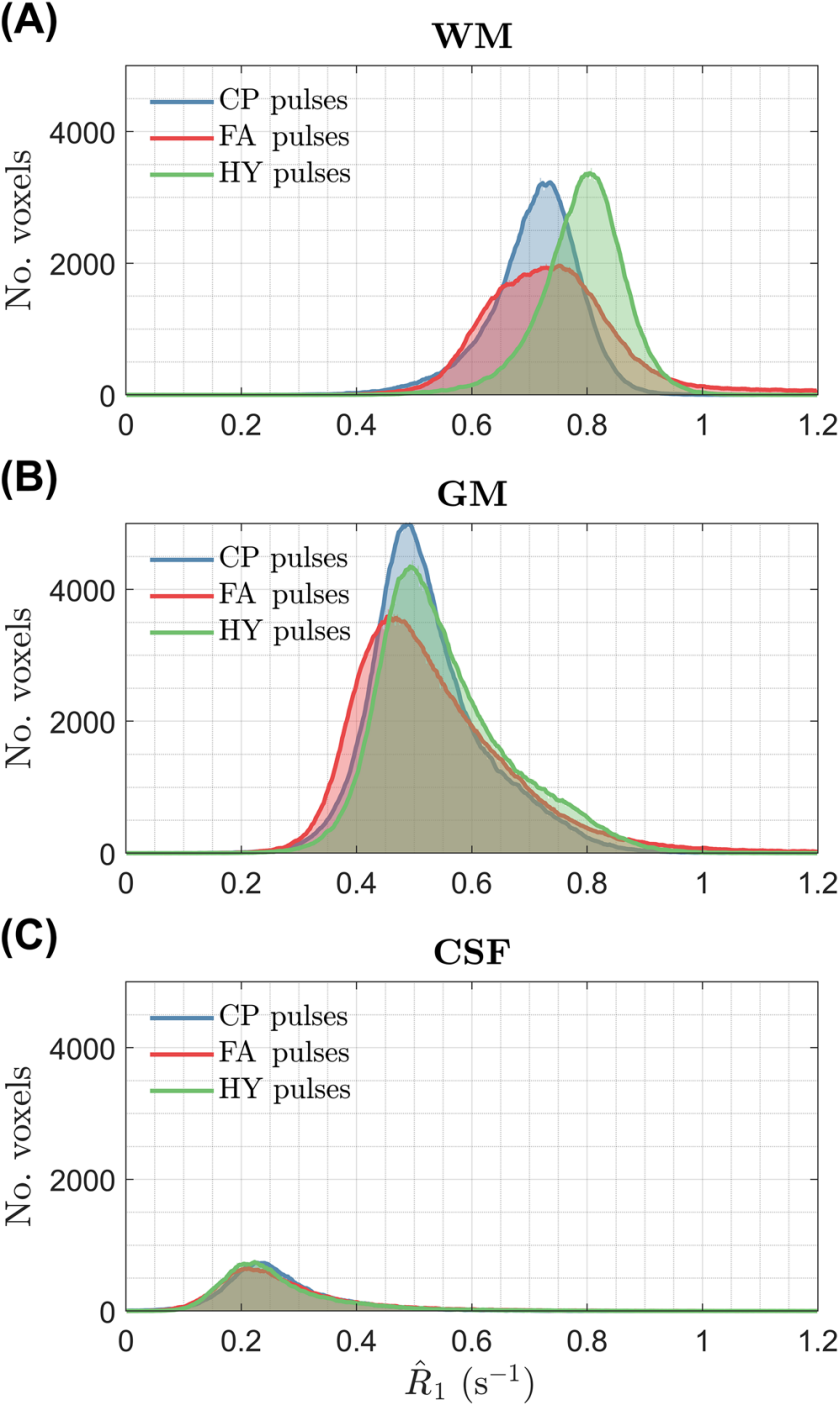
Histograms of the ${\hat{R}}_{1}$ distribution from subject B in (A) white matter, (B) gray matter and (C) CSF for three pulse types: circular polarized (CP), flip angle (FA), and hybrid (HY).

Figure 5 shows the ${\hat{R}}_{1}$ maps for one subject estimated using different pulses; the corresponding α and β maps are given in Figure S3 and the ${\hat{R}}_{1}$ maps for all subjects are in Figure S4. With CP mode the ${\hat{R}}_{1}$ values are fairly uniform, but noisy in the center (green arrow; see zoom‐ins), corresponding to the area of higher α and β intensity. The noisy ${\hat{R}}_{1}$ estimation is no longer present with the FA pulses, but severe shading is observed (blue arrows). Finally, using HY pulses ${\hat{R}}_{1}$ estimation stays precise and no shading is observed. The histograms of the ${\hat{R}}_{1}$ distribution in Figure 6 show tall and narrower distributions in WM and GM with CP and HY pulses, as well as similar mean ${\hat{R}}_{1}$ in GM, but HY pulses having a higher mean ${\hat{R}}_{1}$ in WM. Moreover, FA pulses cause a broader ${\hat{R}}_{1}$ distribution in WM and GM, with mean ${\hat{R}}_{1}$ also different from the other pulses. In CSF, all pulse types show similar mean and dispersion of ${\hat{R}}_{1}$.

**FIGURE 7:**
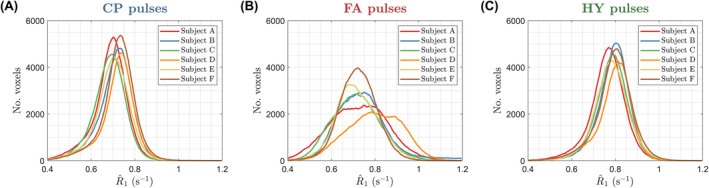
${\hat{R}}_{1}$ distribution in white matter for all subjects using (A) circular polarized (CP) pulses, (B) flip angle (FA) pulses, and (C) hybrid (HY) pulses.

**FIGURE 8:**
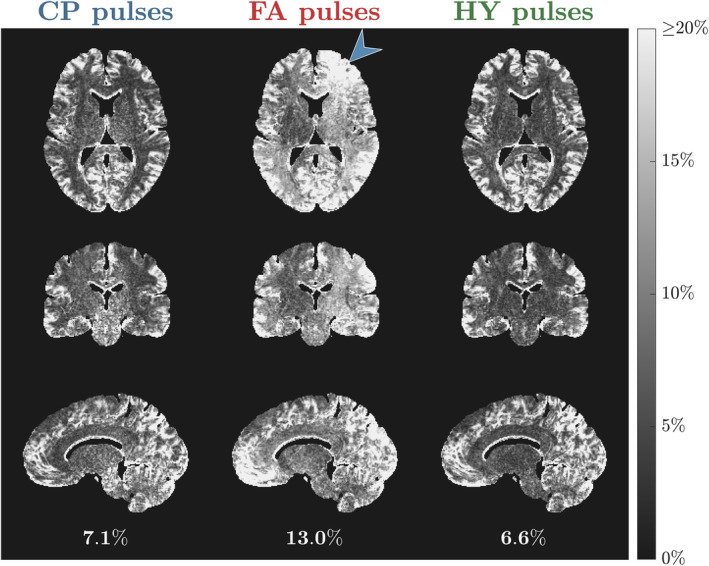
Coefficient of variation (CoV) of the normalized ${\hat{R}}_{1}$ maps across all subjects for each pulse type: circular polarized (CP) pulses (left column), flip angle (FA) pulses (middle column) and hybrid (HY) pulses (right column). Below each sagittal slice is the average CoV of ${\hat{R}}_{1}$ in white matter (WM) for the respective pulse type.

The WM distribution of ${\hat{R}}_{1}$ for all subjects is in Figure 7. Both CP and HY pulses show consistent ${\hat{R}}_{1}$ distributions across all subjects, with mean ${\hat{R}}_{1}$ being smaller for CP pulses, whereas FA pulses exhibit a big variability in the location and dispersion of the distributions. The CoV of ${\hat{R}}_{1}$ across subjects is shown in Figure 8 after diffeomorphic registration of all maps to the MNI space. The CP and HY pulses exhibit a smaller ${\hat{R}}_{1}$ CoV in WM, whereas FA pulses have the largest CoV that changes over the WM (blue arrow) because of different shadings across subjects.

**FIGURE 9:**
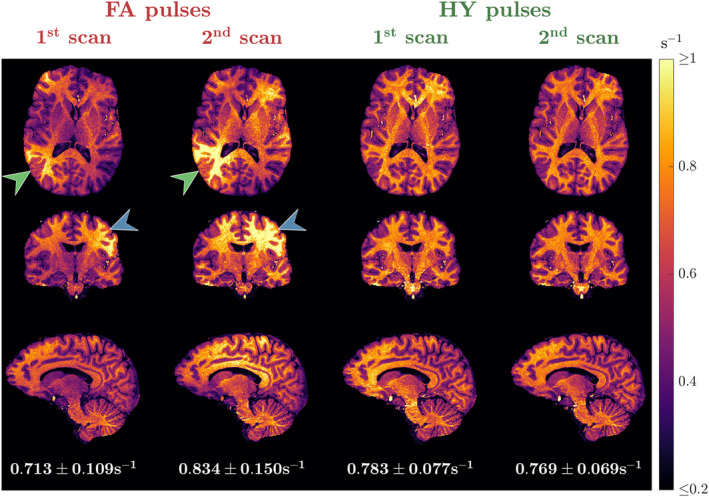
${\hat{R}}_{1}$ maps from two independent scans for one subject using flip angle (FA) pulses (left) and hybrid (HY) pulses (right). The average ± SD of ${\hat{R}}_{1}$ in white matter (WM) is shown below the sagittal slice of each scan. Different pulses were used in each scan. The maps shown belong to subject C.

**FIGURE 10:**
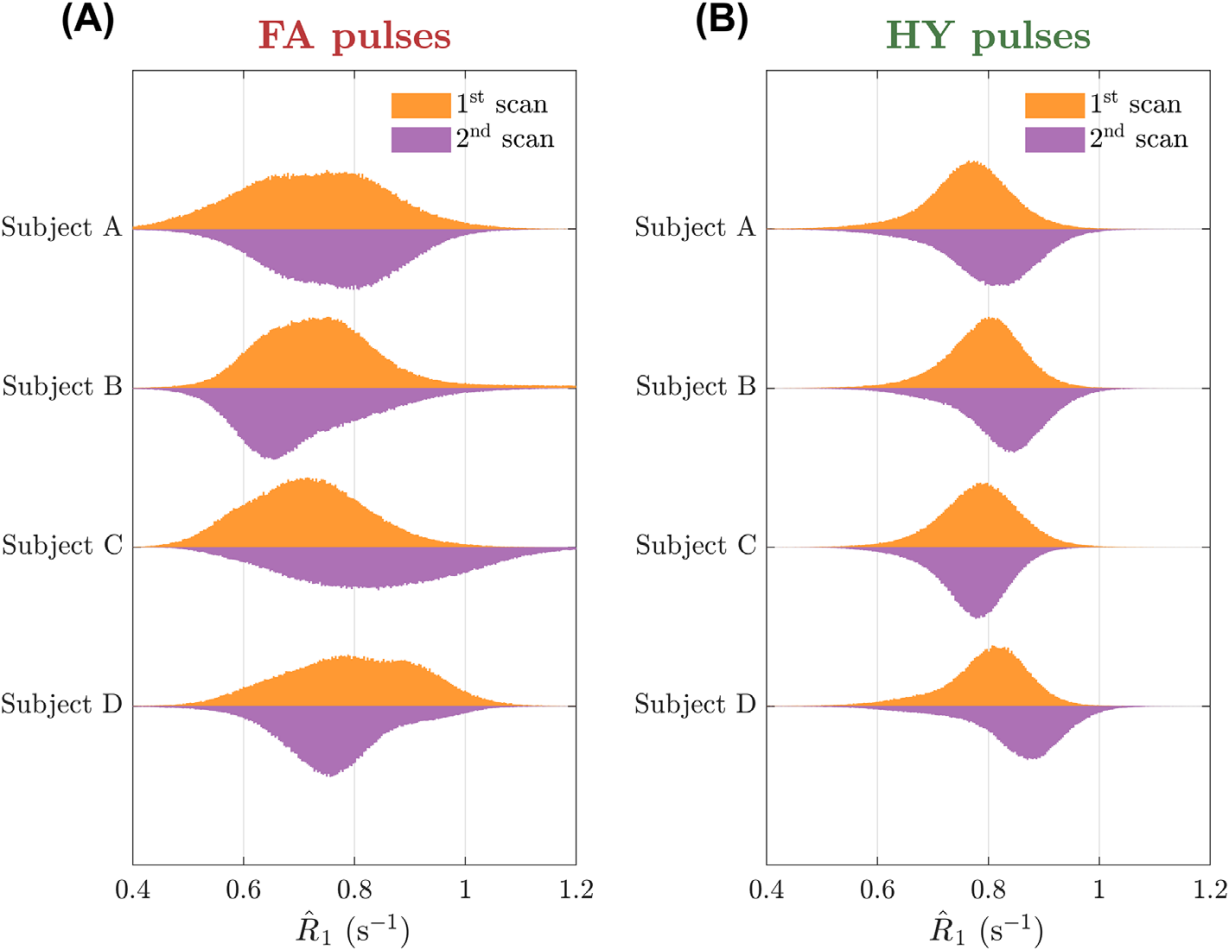
Dual‐sided violin plots of the ${\hat{R}}_{1}$ distribution in white matter (WM) for the first scan (top side of the violin) and the second scan (bottom side of the violin) for four subjects. Distributions obtained with (A) flip angle (FA) pulses and (B) hybrid (HY) pulses.

The ${\hat{R}}_{1}$ maps from subject C for the scan‐rescan test are in Figure 9. With FA pulses the ${\hat{R}}_{1}$ maps obtained in the two scans show considerable differences as indicated by the green and blue arrows, resulting in a different mean ${\hat{R}}_{1}$ in WM (0.713s^−1^ vs. 0.834s^−1^). Moreover, HY pulses yielded similar ${\hat{R}}_{1}$ maps in the two scans, with both looking equally uniform and practically the same mean ${\hat{R}}_{1}$ in WM (0.783s^−1^ vs. 0.769s^−1^). Figure 10 shows the ${\hat{R}}_{1}$ distributions in WM for all subjects across the two scans. Overall, FA designs yield a different distribution when scanning the same subject with new pulses and that is non‐unimodal in some cases. When using different HY pulses the distributions are unimodal and very close to each other. In three of four cases, we observed a shift toward larger ${\hat{R}}_{1}$ values observed in the second HY pulse scan, which was performed later during each scanning session.

## 5 DISCUSSION

This work presents a novel pTx pulse design that simultaneously controls the spatial distributions of flip angle and $B_{1}^{\mathrm{rms}}$ produced by an RF pulse. These dictate the rotation of the free‐water magnetization and the saturation of the semisolid magnetization, respectively, being relevant to applications affected directly or indirectly by MT effects. Here, we showed how the proposed design improves consistency of $R_{1}$ mapping at 7T by controlling incidental MT effects.

Although RF shimming is a straightforward solution to homogenize both flip angle (α) and $B_{1}^{\mathrm{rms}}$ (β) distributions by targeting $B_{1}^{+}$ (or $\left|B_{1}^{+}\right|$),^12^, ^13^ performance for full 3D coverage using our 8‐channel Tx head coil is often similar to that of CP mode^11^ (e.g., Figure S1). Current pTx pulse designs overcome this limitation by instead targeting the flip angle or $B_{1}^{\mathrm{rms}}$ distribution produced by an RF pulse, but not both—this results in a wide distribution of the disregarded property as shown in Figure 4. Although for off‐resonance saturation pulses it is sufficient to consider the distribution of $B_{1}^{\mathrm{rms}}$, but not flip angle variation (as in the PUSH method^24^), for on‐resonance pulses both properties are important in determining the magnetization dynamics of systems with MT. The proposed HY design is a generalization of conventional pulse designs that only consider α and the PUSH design^24^ that only considers β by performing a dual cost function optimization.

### 5.1 HY optimization performance

The HY optimization introduces two new parameters compared to a FA optimization: (1) $\beta_{\mathrm{des}}$, the target $B_{1}^{\mathrm{rms}}$; and (2) λ, the balancing parameter. Theory shows that a lower bound on β can be derived based on the desired on‐resonance flip angle $\alpha_{\mathrm{des}}$. Simulation results in Figure 3 show that for HY optimizations (λ∈(0,1)) the NRMSE of α and β reduce considerably when increasing $\beta_{\mathrm{des}}$ until $\approx 1.2\beta_{\min}$, after which it plateaus until $\beta_{\mathrm{des}}$ reaches the SAR limits and then the NRMSE of β increases sharply. In this case, the NRMSE of α does not increase because its target $\alpha_{\mathrm{des}}$ is achievable within $\beta_{\mathrm{des}}$, that is, $\beta_{\mathrm{des}}>\beta_{\min}$. The threshold $\approx 1.2\beta_{\min}$ is likely to depend on several factors (e.g., coil properties) the volume being imaged and specific properties of the pulse design.

Simulations showed that the pulse design appears to be insensitive to balancing parameter λ, with the NRMSE of α and β only changing considerably when λ→1 and λ→0, respectively. This implies that the optimization finds solutions with simultaneously uniform α and β without having to perform substantial trade‐offs; therefore, an even balancing λ=0.5 was used for the experiments. The insensitivity to λ meant that most of the points in the Pareto front (Figure 3A) were concentrated in a corner, resembling the L‐curve behavior seen in other regularized optimizations.^34^ The local SAR was also found be largely insensitive to λ when this is >0, depending mostly on $\beta_{\mathrm{des}}$ (results not shown).

The performance of the proposed design in 3D is mostly limited by the β optimization. This was observed in the PUSH study^24^ that also reported worse homogeneity in 3D imaging because of the limited variability in the head‐foot direction provided by the coil used. Although for flip angle optimization this can be mitigated by using gradient encoding, β only depends on the transmit sensitivities and RF waveforms. Nevertheless, this key difference provides greater control in achieving both $\alpha_{\mathrm{des}}$ and $\beta_{\mathrm{des}}$ targets as gradients are a degree of freedom that differentiate the two.

### 5.2 DFA $R_{1}$ mapping

The HY pulse design was used for $R_{1}$ mapping with the DFA method that is known to be sensitive to incidental MT effects.^28^, ^29^, ^30^, ^31^ Although flip angle inhomogeneities can be corrected in the fit (Eq. [13]) and cause “noisy”/imprecise estimation because of ill‐conditioned fitting, incidental MT effects created by β cannot be corrected in single pool VFA experiments and cause bias.^28^, ^29^, ^30^, ^31^ The impact of flip angle inhomogeneity was observed in Figure 5 where ${\hat{R}}_{1}$ was noisy in the central regions with CP pulses, which disappeared when using FA and HY pulses. However, the inhomogeneous β produced by the FA pulses introduced severe spatial inhomogeneity of ${\hat{R}}_{1}$. By homogenizing β with the HY pulses the shading was suppressed while preserving a precise estimation because of a homogeneous α. These findings were verified in the CoV of the normalized ${\hat{R}}_{1}$ maps across all subjects (Figure 8), with HY pulses having a small SD similar to that of CP pulses, and last, FA pulses exhibiting the largest variability because of different shadings among subjects. The WM distribution of ${\hat{R}}_{1}$ in Figure 7 also showed that FA pulses yielded the least consistent distributions across all subjects, whereas CP and HY pulses gave very similar distributions. This can be explained by the fact that both CP and HY pulses deliver a similar β for each subject, whereas with FA pulses this can change as is not controlled by the optimization.

The $R_{1}$ mapping reproducibility improved substantially when using HY pulses (Figure 9). Both sets of HY pulses yielded similar ${\hat{R}}_{1}$ maps, whereas with FA pulses test–retest ${\hat{R}}_{1}$ maps showed large differences in the estimated values. For all subjects the WM distribution of ${\hat{R}}_{1}$ changed considerably between the two scans with FA pulses, but retained a consistent unimodal distribution with HY pulses. However, with the latter there was generally a shift in ${\hat{R}}_{1}$ toward longer values in the second scan. A separate phantom experiment revealed some small deviations in the amplitude of the RF power amplifiers (RFPA) (Figure S5) over time that could explain this shift because (by definition) the second scan was always performed later in the scanning session. We and others have reported some instability relating to RF power drifting specifically on this RFPA model and generation^24^, ^41^— 20 seconds of dummycycles were used to minimize this effect as far as possible, but it seems this was not a complete solution.

Overall, the results indicated that homogenizing α with traditional FA pulse design led to worse ${\hat{R}}_{1}$ maps compared to CP pulses by introducing a strong shading across the maps. The HY design solves this problem by homogenizing β, which homogenizes the MT bias and, therefore, results in uniform ${\hat{R}}_{1}$ maps. Interestingly, despite β produced by CP mode being inhomogeneous, ${\hat{R}}_{1}$ maps using this mode showed less bias than FA design. One possible reason was that the CP pulses applied here were shorter and did not involve gradients blips compared to the FA and HY k_T_‐points pulses that could make them less sensitive to errors in the $B_{1}^{+}$ and $B_{0}$ maps^53^ as well as eddy currents.^54^ VFA experiments are sensitive to other sources of bias, such as incomplete spoiling.^55^ We sought to minimize this by using the correction factor for proposed by Baudrexel et al.,^39^ and reduced scanning time to mitigate motion. Nevertheless, any intra‐scan and inter‐scan motion can affect $R_{1}$ mapping by changing the receive sensitivity,^56^ but also the $B_{1}^{+}$ and $B_{0}$ fields,^57^ which in turn changes the α and β maps produced by the RF pulses, and these effects would be less predictable for the pTx methods used than CP mode.

This said, we also believe that the relatively good performance of CP mode compared with the pTx methods in our results is likely down to the choice of flip angles and how the signal biases appearing in each of the two separate component images interacted to cause a bias in the measured $R_{1}$. To test this we conducted an experiment using other nominal flip angles, which yielded less self‐consistent ${\hat{R}}_{1}$ maps when using CP mode (Figure S6). Ultimately there will be circumstances in which CP mode can give reasonable results, and pTx is not necessary; this is a matter that must be addressed on a study specific basis. However, our results show that if a pTx design is used, then the presented HY method provides an effective means for mitigating incidental MT effects, and these effects can result in quite severe artefacts if using more standard flip angle based design methods.

### 5.3 Assumptions, extensions, and other applications

The impact of homogenizing β is smaller than homogenizing α because of (1) the semisolid magnetization being a fraction of the free‐water magnetization; and (2) the semisolid magnetization having virtually no transverse magnetization to create signal, so it is only via exchange with the free‐water magnetization that it produces a visible effect. Nevertheless, this can be important for quantitative methods like MPM,^58^ quantitative MT,^25^ and IR based $R_{1}$ mapping methods^32^, ^59^ where shading in $R_{1}$ mapping have also been observed with MP2RAGE.^60^

Here, we explored DFA $R_{1}$ mapping with each pulse designed to use the smallest reasonable amount of $B_{1}^{\mathrm{rms}}$ ($\beta_{\mathrm{des}}=1.2\beta_{\min}$), but the design could also be used to create the same $B_{1}^{\mathrm{rms}}$ for all pulses as in the controlled saturation MT approach.^30^ Moreover, the design approach can be used generally, both for alternative k‐space trajectories to k_T_‐points (e.g., spiral nonselective,^15^ spokes,^61^, ^62^ etc.) or high tip angle designs such as for inversion or refocusing pulses, as well as for universal pulses.^63^ For the case of high tip angle pulses, longer pulse durations may be necessary such that relaxation and exchange can no longer be neglected. In that scenario, the magnetization response of both pools becomes intertwined, violating the assumption in the PUSH design that the pools are independent during RF exposure. This scenario would require more complex designs that are beyond the scope of this study.

## 6 CONCLUSION

This work proposed a novel HY optimization of RF pulses to homogenize both their flip angle and $B_{1}^{\mathrm{rms}}$ distributions simultaneously. Simulations showed that it is possible to obtain pulses with both uniform flip angle and $B_{1}^{\mathrm{rms}}$ without big detriment of each property compared to an individual optimization. As an exemplar, we applied the approach to single pool $R_{1}$ mapping and showed that the HY optimized pulses produce clearly more uniform estimates than flip angle optimized pulses, because of this effect. In this application, the HY pulse design provides a way to spatially control MT effects, therefore, its bias, to achieve uniform ${\hat{R}}_{1}$ estimation, as well as to control the flip angles that impact estimation precision. The HY design method is general and could be used in other scenarios such as other quantitative imaging methods, or for non‐quantitative sequences where incidental MT effects have a significant effect on contrast.

## FUNDING INFORMATION

National Institute for Health Research (NIHR) Biomedical Research Centre based at Guy's and St Thomas' NHS Foundation Trust and King's College London and/or the NIHR Clinical Research Facility, funded by the King's College London & Imperial College London EPSRC Centre for Doctoral Training in Medical Imaging [EP/L015226/1], by core funding from the Wellcome/EPSRC Centre for Medical Engineering [WT203148/Z/16/Z] and by a Wellcome Trust Collaboration in Science Award [WT201526/Z/16/Z].

## CONFLICT OF INTEREST STATEMENT

R.T.T., P.L., R.G., and D.R. work for Siemens Healthcare.

## Supporting information


**Figure S1.** Results obtained with a magnitude least squares (MLS) RF shimming solution. Left column: polar plot with the amplitude (Volts) and phases (radians) of the MLS RF shim (filled circles), compared to those of the CP pulse (empty circles). Middle column: respective flip angle map. Right column: respective $B_{1}^{\mathrm{rms}}$ map.
**Figure S2.** Surfaces of the two terms in the pulse design cost function: (A) NRMSE of the flip angle and (B) NRMSE of the $B_{1}^{\mathrm{rms}}$, for all optimized values of λ and $\beta_{\mathrm{des}}$.
**Figure S3.** Flip angle (left column) and $B_{1}^{\mathrm{rms}}$ (right column) maps of each pulse type (CP, FA optimized, HY optimized) for the two SPGRs acquired to estimate the ${\hat{R}}_{1}$ map in Figure 5. Top and bottom rows correspond to the SPGRs acquired with $\alpha_{\mathrm{des}}$ = 3° and $\alpha_{\mathrm{des}}$ = 15°, respectively.
**Figure S4.** Sagittal view of the ${\hat{R}}_{1}$ maps acquired with CP, FA and HY pulses (top, middle and bottom rows respectively) for all subjects (left to right: subject A, B, C, D, E and F). To navigate through different slices this document needs to be open on a JavaScript‐supporting PDF viewer, such as Adobe Acrobat Reader.
**Figure S5.** Variation of the (A) amplitude and (B) phase of the 5 sub‐pulses in the same k_T_‐points pulse over time relative to the 1st scan, where the traces were measured using the directional couplers (DICOs). The bar range for each point represents the standard deviation across the 5 sub‐pulses. The dashed lines in (B) represent the phase drift with respect to the average of all channels.
**Figure S6.** ${\hat{R}}_{1}$ maps obtained with CP pulses for different combinations of flip angles: columns are different T1‐weighted images, from $\alpha_{\mathrm{nom}}$ = 12° (left), $\alpha_{\mathrm{nom}}$ = 15° (middle), to $\alpha_{\mathrm{nom}}$ = 18° (right); rows are different PD‐weighted images, from $\alpha_{\mathrm{nom}}$ = 2° (top) to $\alpha_{\mathrm{nom}}$ = 4° (bottom). Below each sagittal slice is the average ± standard deviation of ${\hat{R}}_{1}$ in WM for the respective combination of flip angles.

## Data Availability

According to United Kingdom research councils' Common Principles on Data Policy and Wellcome Trust's Policy on data, software and materials management and sharing, all simulated data supporting this study is openly available at https://github.com/mriphysics/HybridPTx (hash 98fc91d at time of submission). This will exclude proprietary code from SIEMENS, but that can be shared on request by agreement including the vendor, and in vivo MRI data because of the terms of the ethical approval under which they were acquired.

## References

[1] Lüsebrink F , Mattern H , Yakupov R , et al. Comprehensive ultrahigh resolution whole brain in vivo MRI dataset as a human phantom. Sci Data. 2021;8:138. 34035308 10.1038/s41597-021-00923-w PMC8149725

[2] Wang F , Dong Z , Tian Q , et al. In vivo human whole‐brain Connectom diffusion MRI dataset at 760 μm isotropic resolution. Sci Data. 2021;8:122. 33927203 10.1038/s41597-021-00904-z PMC8084962

[3] Van De Moortele PF , Akgun C , Adriany G , et al. B1 destructive interferences and spatial phase patterns at 7 T with a head transceiver array coil. Magn Reson Med. 2005;54:1503‐1518. 16270333 10.1002/mrm.20708

[4] Collins CM , Liu W , Schreiber W , Yang QX , Smith MB . Central brightening due to constructive interference with, without, and despite dielectric resonance. J Magn Reson Imaging. 2005;21:192‐196. 15666397 10.1002/jmri.20245

[5] Tannús A , Garwood M . Adiabatic pulses. NMR Biomed. 1997;10:423‐434. 9542739 10.1002/(sici)1099-1492(199712)10:8<423::aid-nbm488>3.0.co;2-x

[6] Yang QX , Mao W , Wang J , et al. Manipulation of image intensity distribution at 7.0T: Passive RF shimming and focusing with dielectric materials. J Magn Reson Imaging. 2006;24:197‐202. 16755543 10.1002/jmri.20603

[7] Katscher U , Börnert P , Leussler C , Van den Brink JS . Transmit sense. Magn Reson Med. 2003;49:144‐150. 12509830 10.1002/mrm.10353

[8] Zhu Y . Parallel excitation with an array of transmit coils. Magn Reson Med. 2004;51:775‐784. 15065251 10.1002/mrm.20011

[9] Hoult DI , Phil D . Sensitivity and power deposition in a high‐field imaging experiment. J Magn Reson Imaging. 2000;12:46‐67. 10931564 10.1002/1522-2586(200007)12:1<46::aid-jmri6>3.0.co;2-d

[10] Padormo F , Beqiri A , Hajnal JV , Malik SJ . Parallel transmission for ultrahigh‐field imaging. NMR Biomed. 2016;29:1145‐1161. doi:10.1002/nbm.3313 25989904 PMC4995736

[11] Mao W , Smith MB , Collins CM . Exploring the limits of RF shimming for high‐field MRI of the human head. Magn Reson Med. 2006;56:918‐922. 16958070 10.1002/mrm.21013 PMC4040521

[12] Setsompop K , Wald LL , Alagappan V , Gagoski BA , Adalsteinsson E . Magnitude least squares optimization for parallel radio frequency excitation design demonstrated at 7 tesla with eight channels. Magn Reson Med. 2008;59:908‐915. 18383281 10.1002/mrm.21513 PMC2715966

[13] Katscher U , Vernickel P , Graesslin I , Börnert P . RF shimming using a multi‐element transmit system in phantom and in vivo studies. Proc Intl Soc Mag Reson Med. 2007;15:1693.

[14] Cloos MA , Boulant N , Luong M , et al. kT‐points: short three‐dimensional tailored RF pulses for flip‐angle homogenization over an extended volume. Magn Reson Med. 2012;67:72‐80. 21590724 10.1002/mrm.22978

[15] Malik SJ , Keihaninejad S , Hammers A , Hajnal JV . Tailored excitation in 3D with spiral nonselective (SPINS) RF pulses. Magn Reson Med. 2012;67:1303‐1315. 21842503 10.1002/mrm.23118

[16] Cao Z , Donahue MJ , Ma J , Grissom WA . Joint design of large‐tip‐angle parallel RF pulses and blipped gradient trajectories. Magn Reson Med. 2016;75:1198‐1208. 25916408 10.1002/mrm.25739 PMC4624053

[17] Bloch F . Nuclear induction. Phys Rev. 1946;70:460‐474.

[18] Orzylowska A , Slowik T , Chudzik A , Pankowska A , Lam W , Stanisz G . The CLARITY procedure of lipid removal from brain tissue sample reveals the lipid‐origin of MT contrast in CEST imaging experiment. Proceedings of the 28th Annual Meeting of ISMRM. ISMRM; 2020:497.

[19] Assländer J , Gultekin C , Flassbeck S , Glaser SJ , Sodickson DK . Generalized Bloch model: a theory for pulsed magnetization transfer. Magn Reson Med. 2022;87:2003‐2017. 34811794 10.1002/mrm.29071 PMC8810695

[20] Henkelman RM , Huang X , Xiang QS , Stanisz GJ , Swanson SD , Bronskill MJ . Quantitative interpretation of magnetization transfer. Magn Reson Med. 1993;29:759‐766. 8350718 10.1002/mrm.1910290607

[21] Graham SJ , Henkelman RM . Understanding pulsed magnetization transfer. J Magn Reson Imaging. 1997;7:903‐912. 9307918 10.1002/jmri.1880070520

[22] Balaban RS , Ceckler TL . Magnetization transfer contrast in magnetic resonance imaging. Magn Reson Q. 1992;8:116‐137. 1622774

[23] Henkelman RM , Stanisz GJ , Graham SJ . Magnetization transfer in MRI: a review. NMR Biomed. 2001;14:57‐64. 11320533 10.1002/nbm.683

[24] Leitão D , Tomi‐Tricot R , Bridgen P , et al. Parallel transmit pulse design for saturation homogeneity (PUSH) for magnetization transfer imaging at 7T. Magn Reson Med. 2022;88:180‐194. 35266204 10.1002/mrm.29199 PMC9315051

[25] Dortch RD , Moore J , Li K , et al. Quantitative magnetization transfer imaging of human brain at 7T. Neuroimage. 2013;64:640‐649. 22940589 10.1016/j.neuroimage.2012.08.047 PMC3625658

[26] Soustelle L , Troalen T , Hertanu A , et al. Quantitative magnetization transfer MRI unbiased by on‐resonance saturation and dipolar order contributions. Magn Reson Med. 2023;90:875‐893. doi:10.1002/mrm.29678 37154400

[27] Bagnato F , Hametner S , Franco G , et al. Selective inversion recovery quantitative magnetization transfer brain MRI at 7T: clinical and postmortem validation in multiple sclerosis. J Neuroimaging. 2018;28:380‐388. 29676026 10.1111/jon.12511 PMC6030463

[28] Olsson H , Andersen M , Lätt J , Wirestam R , Helms G . Reducing bias in dual flip angle T1‐mapping in human brain at 7T. Magn Reson Med. 2020;84:1347‐1358. 32060952 10.1002/mrm.28206

[29] Teixeira RP , Neji R , Wood TC , Baburamani AA , Malik SJ , Hajnal JV . Controlled saturation magnetization transfer for reproducible multivendor variable flip angle T_1_ and T_2_ mapping. Magn Reson Med. 2020;84:221‐236. doi:10.1002/mrm.28109 31846122 PMC7154666

[30] Teixeira RPAG , Malik SJ , Hajnal JV . Fast quantitative MRI using controlled saturation magnetization transfer. Magn Reson Med. 2019;81:907‐920. 30257044 10.1002/mrm.27442 PMC6492254

[31] Ou X , Gochberg DF . MT effects and T1 quantification in single‐slice spoiled gradient echo imaging. Magn Reson Med. 2008;59:835‐845. 18302249 10.1002/mrm.21550 PMC4186261

[32] Rioux JA , Levesque IR , Rutt BK . Biexponential longitudinal relaxation in white matter: characterization and impact on T_1_ mapping with IR‐FSE and MP2RAGE. Magn Reson Med. 2016;75:2265‐2277. 26190230 10.1002/mrm.25729 PMC4837078

[33] Weigel M , Helms G , Hennig J . Investigation and modeling of magnetization transfer effects in two‐dimensional multislice turbo spin echo sequences with low constant or variable flip angles at 3 T. Magn Reson Med. 2010;63:230‐234. 19859950 10.1002/mrm.22145

[34] Grissom W , Yip CY , Zhang Z , Stenger VA , Fessler JA , Noll DC . Spatial domain method for the design of RF pulses in multicoil parallel excitation. Magn Reson Med. 2006;56:620‐629. 16894579 10.1002/mrm.20978

[35] Pauly J , Nishimura D , Macovski A . A k‐space analysis of small‐tip‐angle excitation. J Magn Reson (1969). 1989;81:43‐56. doi:10.1016/0022-2364(89)90265-5 22152370

[36] Eichfelder G , Gebhardt M . Local specific absorption rate control for parallel transmission by virtual observation points. Magn Reson Med. 2011;66:1468‐1476. 21604294 10.1002/mrm.22927

[37] Fram EK , Herfkens RJ , Johnson GA , et al. Rapid calculation of T1 using variable flip angle gradient refocused imaging. Magn Reson Imaging. 1987;5:201‐208. 3626789 10.1016/0730-725x(87)90021-x

[38] Wood TC . Improved formulas for the two optimum VFA flip‐angles. Magn Reson Med. 2015;74:1‐3. 25754124 10.1002/mrm.25592

[39] Baudrexel S , Nöth U , Schüre JR , Deichmann R . T_1_ mapping with the variable flip angle technique: a simple correction for insufficient spoiling of transverse magnetization. Magn Reson Med. 2018;79:3082‐3092. 29052267 10.1002/mrm.26979

[40] Griswold MA , Jakob PM , Heidemann RM , et al. Generalized autocalibrating partially parallel acquisitions (GRAPPA). Magn Reson Med. 2002;47:1202‐1210. 12111967 10.1002/mrm.10171

[41] Aghaeifar A , Bosch D , Heule R , et al. Intra‐scan RF power amplifier drift correction. Magn Reson Med. 2024;92:645‐659. doi:10.1002/mrm.30078 38469935

[42] Jenkinson M , Smith S . A global optimisation method for robust affine registration of brain images. Med Image Anal. 2001;5:143‐156. 11516708 10.1016/s1361-8415(01)00036-6

[43] Marques JP , Kober T , Krueger G , van der Zwaag W , van de Moortele PF , Gruetter R . MP2RAGE, a self bias‐field corrected sequence for improved segmentation and T1‐mapping at high field. Neuroimage. 2010;49:1271‐1281. doi:10.1016/j.neuroimage.2009.10.002 19819338

[44] Ashburner J , Friston KJ . Unified segmentation. Neuroimage. 2005;26:839‐851. 15955494 10.1016/j.neuroimage.2005.02.018

[45] Yarnykh VL . Actual flip‐angle imaging in the pulsed steady state: a method for rapid three‐dimensional mapping of the transmitted radiofrequency field. Magn Reson Med. 2007;57:192‐200. 17191242 10.1002/mrm.21120

[46] Nehrke K . On the steady‐state properties of actual flip angle imaging (AFI). Magn Reson Med. 2009;61:84‐92. 19097210 10.1002/mrm.21592

[47] Van De Moortele P‐F , Snyder C , DelaBarre L , Adriany G , Vaughan JT , Ugurbil K . Calibration tools for RF shim at very high field with multiple element RF coils: from ultra fast local relative phase to absolute magnitude B1+ mapping. Proceedings of the **International Society** for **Magnetic Resonance** in **Medicine** . Vol 15. ISMRM; 2007:1676.

[48] Ashburner J . A fast diffeomorphic image registration algorithm. Neuroimage. 2007;38:95‐113. 17761438 10.1016/j.neuroimage.2007.07.007

[49] Tabelow K , Balteau E , Ashburner J , et al. hMRI – a toolbox for quantitative MRI in neuroscience and clinical research. Neuroimage. 2019;194:191‐210. 30677501 10.1016/j.neuroimage.2019.01.029 PMC6547054

[50] IEC‐60601‐2‐33 . Medical Electrical Equipment‐Part 2‐33: Particular Requirements for the Basic Safety and Essential Performance of Magnetic Resonance Equipment for Medical Diagnosis, IEC 60601‐2‐33: 2010/AMD2: 2015. 2010.

[51] Tomi‐Tricot R , Sedlacik J , Endres J , et al. Fully integrated scanner implementation of direct signal control for 2D T2‐weighted TSE at ultra‐high field. Proceedings of the **International Society** for **Magnetic Resonance** in **Medicine** . Vol 29. ISMRM; 2021:621.

[52] Smith SM . Fast robust automated brain extraction. Hum Brain Mapp. 2002;17:143‐155. 12391568 10.1002/hbm.10062 PMC6871816

[53] Samsonov AA , Yarnykh VL . Accurate actual flip angle imaging (AFI) in the presence of fat. Magn Reson Med. 2024;91:2345‐2357. doi:10.1002/mrm.30000 38193249 PMC10997465

[54] Boulant N , le Ster C , Amadon A , et al. The possible influence of third‐order shim coils on gradient–magnet interactions: an inter‐field and inter‐site study. MAGMA. 2024;37:169‐183. doi:10.1007/s10334-023-01138-3 38197908 PMC10995016

[55] Corbin N , Callaghan MF . Imperfect spoiling in variable flip angle T_1_ mapping at 7T: quantifying and minimizing impact. Magn Reson Med. 2021;86:693‐708. 33645814 10.1002/mrm.28720 PMC8436769

[56] Balbastre Y , Aghaeifar A , Corbin N , Brudfors M , Ashburner J , Callaghan MF . Correcting inter‐scan motion artifacts in quantitative R1 mapping at 7T. Magn Reson Med. 2022;88:280‐291. 35313378 10.1002/mrm.29216 PMC9314963

[57] Plumley A , Watkins L , Treder M , Liebig P , Murphy K , Kopanoglu E . Rigid motion‐resolved B1+ prediction using deep learning for real‐time parallel‐transmission pulse design. Magn Reson Med. 2022;87:2254‐2270. 34958134 10.1002/mrm.29132 PMC7613077

[58] Weiskopf N , Suckling J , Williams G , et al. Quantitative multi‐parameter mapping of R_1_, PD*, MT, and R2* at 3T: a multi‐center validation. Front Neurosci. 2013;7:95. doi:10.3389/fnins.2013.00095 23772204 PMC3677134

[59] Reynolds LA , Morris SR , Vavasour IM , et al. Nonaqueous magnetization following adiabatic and selective pulses in brain: T1 and cross‐relaxation dynamics. NMR Biomed. 2023;36:e4936. doi:10.1002/nbm.4936 36973767 PMC13587261

[60] Bapst B , Massire A , Mauconduit F , et al. Pushing MP2RAGE boundaries: ultimate time‐efficient parameterization combined with exhaustive T1 synthetic contrasts. Magn Reson Med. 2023;91:1608‐1624. doi:10.1002/mrm.29948 38102807

[61] Saekho S , Yip CY , Noll DC , Boada FE , Stenger VA . Fast‐kz three‐dimensional tailored radiofrequency pulse for reduced B1 inhomogeneity. Magn Reson Med. 2006;55:719‐724. 16526012 10.1002/mrm.20840 PMC3076290

[62] Zelinski AC , Wald LL , Setsompop K , et al. Fast slice‐selective radio‐frequency excitation pulses for mitigating B1+ inhomogeneity in the human brain at 7 Tesla. Magn Reson Med. 2008;59:1355‐1364. 18506800 10.1002/mrm.21585 PMC2723802

[63] Gras V , Vignaud A , Amadon A , Le Bihan D , Boulant N . Universal pulses: a new concept for calibration‐free parallel transmission. Magn Reson Med. 2017;77:635‐643. 26888654 10.1002/mrm.26148

