# Supplementary material for: Parallel transmit hybrid pulse design for controlled on-resonance magnetization transfer in R1 mapping at 7T
Source: Magn Reson Med. Author manuscript; Available in PMC 2025 Mar 1. (PMC11680742; doi:10.1002/mrm.30333)
Supplement: Supporting Information [file EMS201792-supplement-Supporting_Information.pdf]

Supporting Information for parallel transmit hybrid pulse design to control on-resonance magnetization transfer at 7T

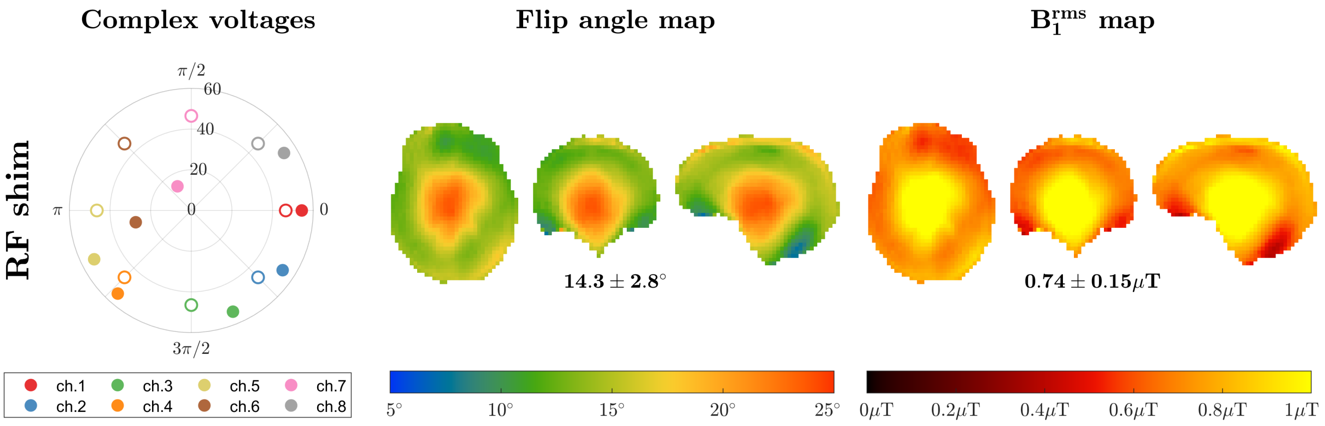

Supporting Information Figure S1: Results obtained with a magnitude least squares (MLS) RF shimming solution. Left column: polar plot with the amplitude (Volts) and phases (radians) of the MLS RF shim (filled circles), compared to those of the CP pulse (empty circles). Middle column: respective flip angle map. Right column: respective B_1^{rms} map.

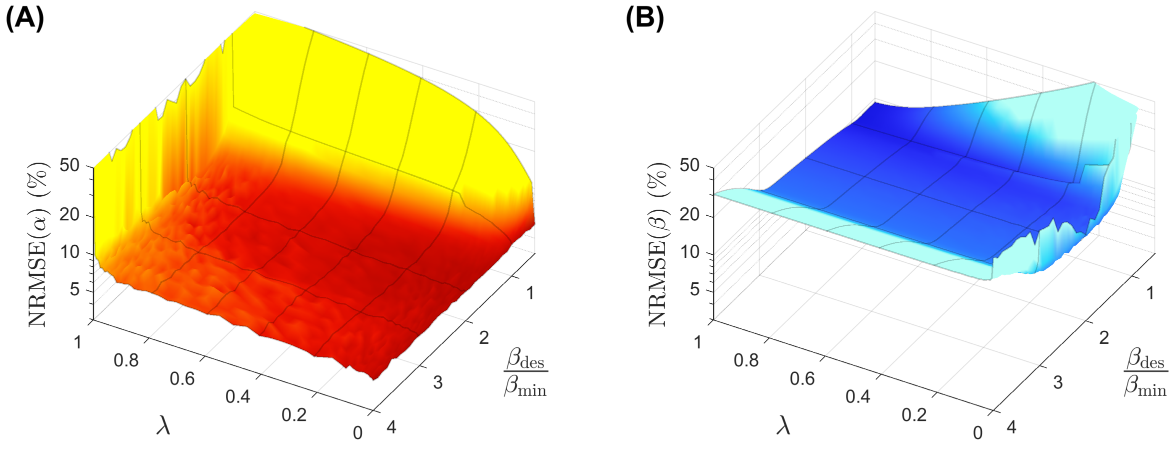

Supporting Information Figure S2: Surfaces of the two terms in the pulse design cost function: (A) NRMSE of the flip angle and (B) NRMSE of the B_1^{rms}, for all optimized values of λ and β_{des}.

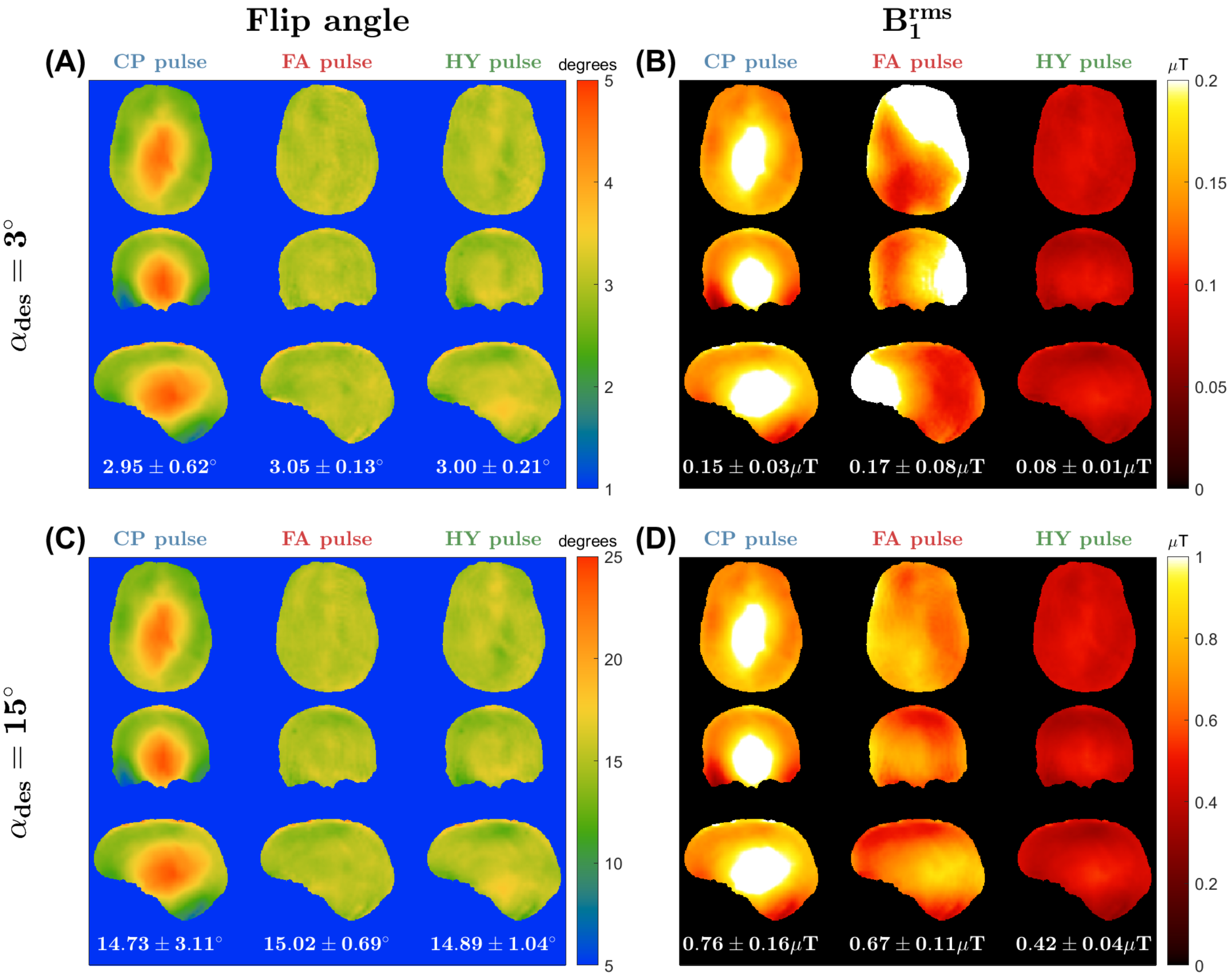

Supporting Information Figure S3: Flip angle (left column) and B_1^{rms} (right column) maps of each pulse type (CP, FA optimized, HY optimized) for the two SPGRs acquired to estimate the $\hat{R}_1$ map in Figure 5. Top and bottom rows correspond to the SPGRs acquired with $\alpha_{des} = 3°$ and $\alpha_{des} = 15°$, respectively.

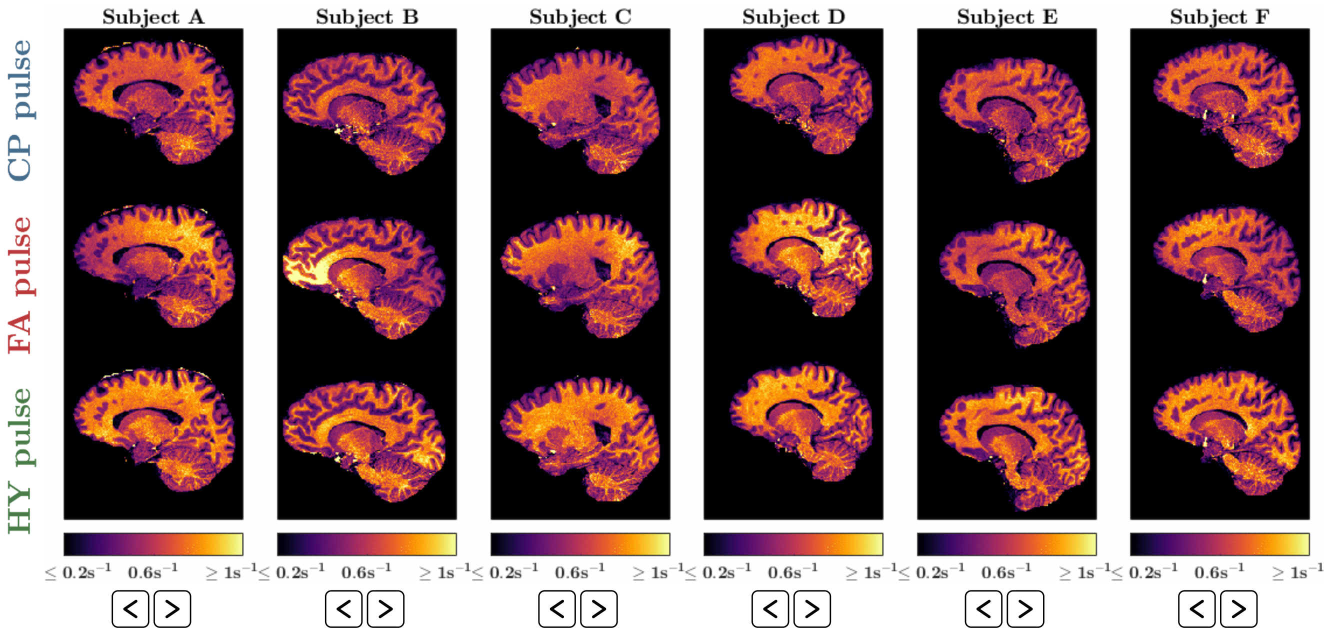

Supporting Information Figure S4: Sagittal view of the $\hat{\mathrm{R}}_1$ maps acquired with CP, FA and HY pulses (top, middle and bottom rows respectively) for all subjects (left to right: subject A, B, C, D, E and F). To navigate through different slices this document needs to be open on a JavaScript-supporting PDF viewer, such as Adobe Acrobat Reader.

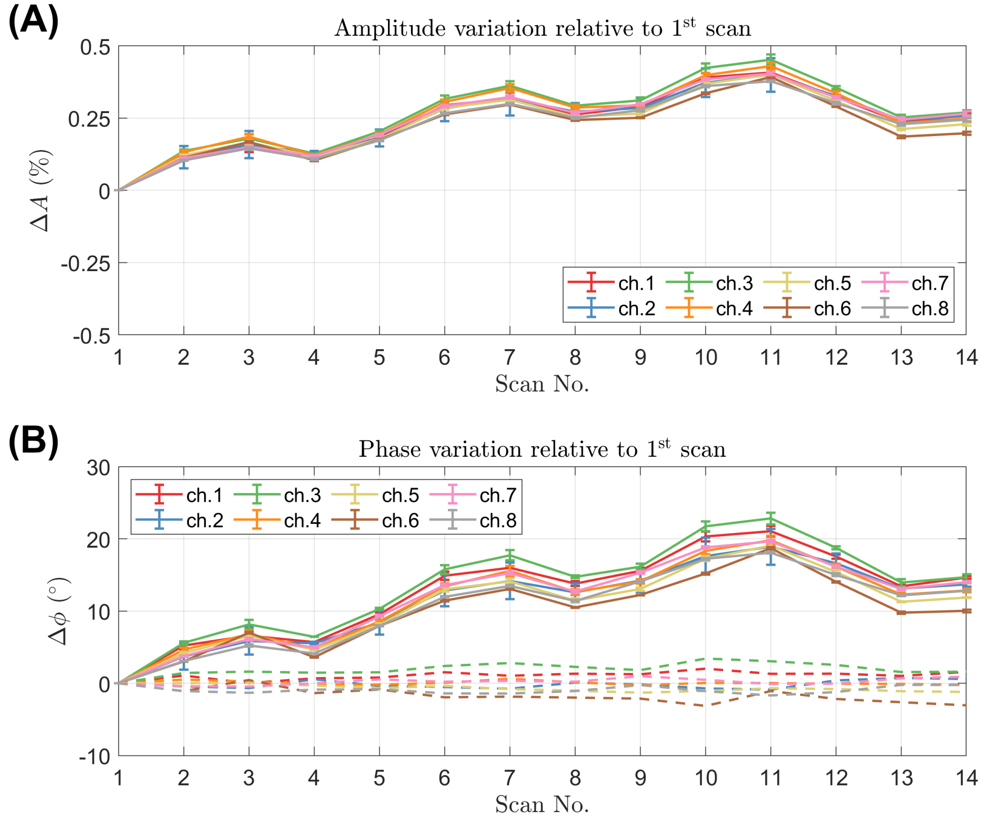

Supporting Information Figure S5: Variation of the (A) amplitude and (B) phase of the 5 sub-pulses in the same $\mathrm{k_T}$-points pulse over time relative to the 1st scan, where the traces were measured using the directional couplers (DICOs). The bar range for each point represents the standard deviation across the 5 sub-pulses. The dashed lines in (B) represent the phase drift with respect to the average of all channels.

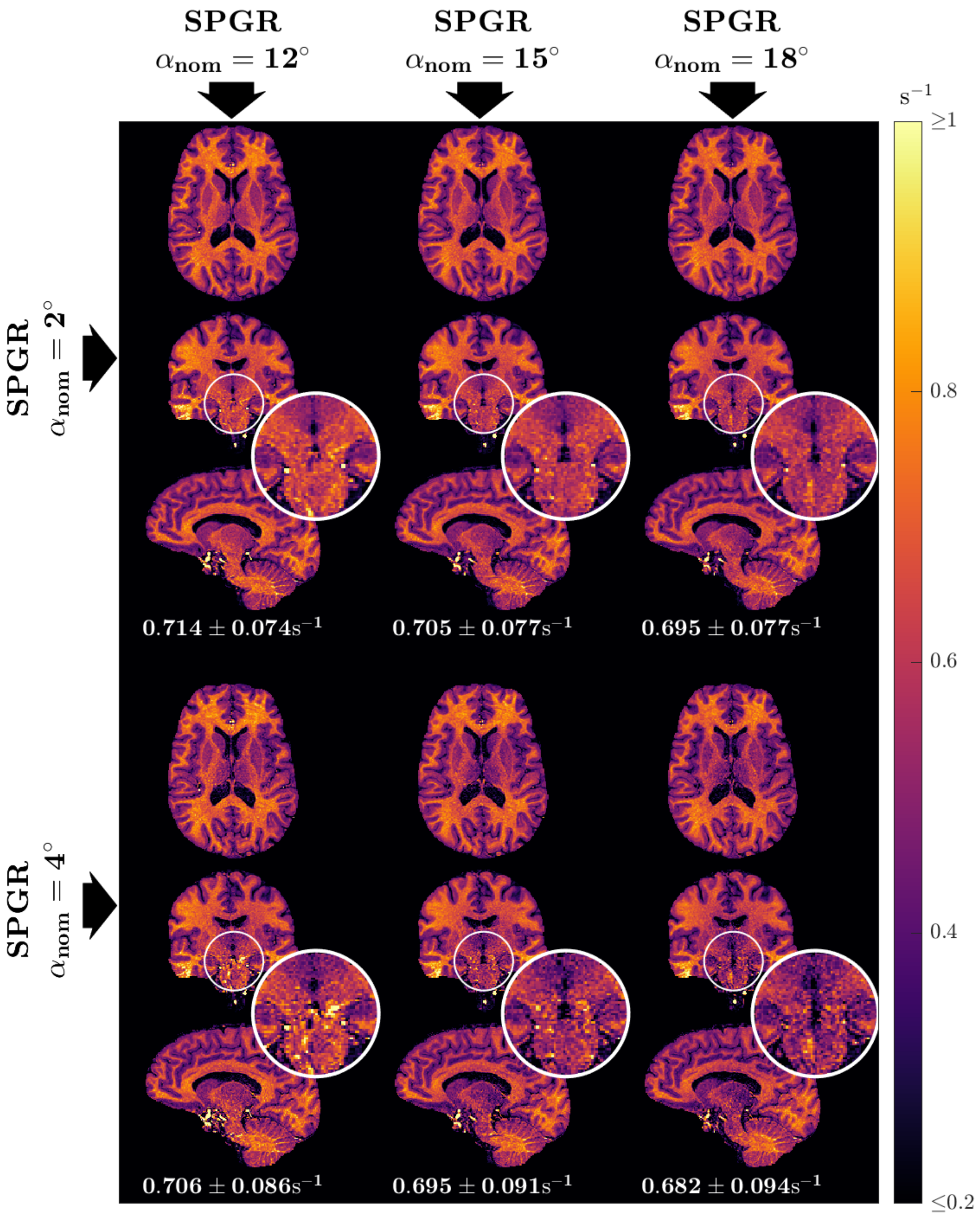

Supporting Information Figure S6: $\hat{R}_1$ maps obtained with CP pulses for different combinations of flip angles: columns are different T_1-weighted images, from $\alpha_{nom} = 12°$ (left), $\alpha_{nom} = 15°$ (middle), to $\alpha_{nom} = 18°$ (right); rows are different PD-weighted images, from $\alpha_{nom} = 2°$ (top) to $\alpha_{nom} = 4°$ (bottom). Below each sagittal slice is the average $\pm$ standard deviation of $\hat{R}_1$ in WM for the respective combination of flip angles.
